# Prediction of Building Limestone Physical and Mechanical Properties by Means of Ultrasonic P-Wave Velocity

**DOI:** 10.1155/2014/508073

**Published:** 2014-01-05

**Authors:** Giovanna Concu, Barbara De Nicolo, Monica Valdes

**Affiliations:** Department of Civil and Environmental Engineering and Architecture, University of Cagliari, Via Marengo 2, 09123 Cagliari, Italy

## Abstract

The aim of this study was to evaluate ultrasonic P-wave velocity as a feature for predicting some physical and mechanical properties that describe the behavior of local building limestone. To this end, both ultrasonic testing and compressive tests were carried out on several limestone specimens and statistical correlation between ultrasonic velocity and density, compressive strength, and modulus of elasticity was studied. The effectiveness of ultrasonic velocity was evaluated by regression, with the aim of observing the coefficient of determination *r* ^2^ between ultrasonic velocity and the aforementioned parameters, and the mathematical expressions of the correlations were found and discussed. The strong relations that were established between ultrasonic velocity and limestone properties indicate that these parameters can be reasonably estimated by means of this nondestructive parameter. This may be of great value in a preliminary phase of the diagnosis and inspection of stone masonry conditions, especially when the possibility of sampling material cores is reduced.

## 1. Introduction

The goal of planning building preservation in the best way has become a very important need over the last decades. Conservation, rehabilitation, and strengthening of the built heritage and protection of human lives are pressing demands of modern societies. Indeed, cultural, social, and economic reasons call for the extension of a building's life beyond its physiological limits. One of the major challenges in the rehabilitation and repair of existing structures is the inspection, which includes detection of damaged zones, cracking and defects, and mechanical characterization of materials. The inspection process becomes particularly complex when dealing with buildings of historical relevance, because evaluation of the actual safety level of the structure should not interfere with its current condition. In this light, it is essential to understand the mechanism of structural deterioration, since its accurate modeling is the most critical component in maintenance management of the built heritage.

In this context, developments in nondestructive evaluation (NDE) tools as methodologies for historical building assessment have grown significantly in recent years, because the use of these inspection techniques allows one to acquire several relevant data without interfering with the state of the asset, thus making it possible to properly plan rehabilitation actions and prepare and manage maintenance budgets. In fact, outputs from inspections and assessments of a structure become inputs in maintenance, rehabilitation, and replacement strategies, with the objectives of ensuring public safety, monitoring structural performance, identifying deficiencies, and thus facilitating immediate intervention.

Several NDE tools are now accessible for building diagnosis, including mechanical, electromagnetic, acoustic, and chemical methods [1–3]. Since the diagnosis of the degradation level depends on damage indicators (crack diffusion, sound velocity, etc.) measured during the inspection and associated with the chosen inspection method, the choice of the diagnostic system most suitable for the actual analysis and the correct interpretation of the survey results become crucial.

As a major NDE tool, acoustic techniques, based on measurements of the characteristics of acoustic waves propagating through the material, are often used in quality control and fault detection in existing buildings. Acoustic material analysis is based on a simple principle of physics: the propagation of any wave will be affected by the medium through which it travels. Thus, changes in measurable parameters associated with the passage of a wave through a material can be correlated with changes in the material's physical properties.

One of the acoustic NDE tools, ultrasonic testing (UT), is based on the study of phenomena connected with the propagation of ultrasonic waves inside the materials under study. The signal that penetrates into the material is generated artificially by an external source and acquired by means of a receiver after passing through the medium following appropriate trajectories. From analyses of the processes and parameters connected with the propagation of the elastic perturbation inside the artifact, it is possible to collect a great deal of information on the material or structure under study [4–11]. In particular, the ultrasonic velocity (*V*) of longitudinal waves has been pointed out by several authors as a useful and reliable nondestructive tool for assessing the physical and mechanical characteristics of materials, such as density, modulus of elasticity, and strength, in both concrete and rock elements [12–18].

UT has obvious advantages compared to traditional invasive tests and it also has several advantages compared to other NDE tools: (i) sensitiveness to both surface and subsurface discontinuities; (ii) depth of penetration superior to other NDE tools such as infrared thermography; (iii) no hazard to operator or nearby personnel and no influence on the material being tested; (iv) instantaneous results provided by electronic equipment; (v) detailed images produced with automated systems; (vi) highly portable or highly automated equipment.

When applying UT, a signal of frequency higher than 20 kHz is transmitted through the material. The conversion of electrical pulses to mechanical vibrations and the conversion of returned mechanical vibrations back into electrical energy are generally performed by using piezoelectric transducers. Parameters associated with the signal penetrating through the medium and taken into account in the analysis are as follows:

- transit time (or travel time), that is, the time taken by the signal to cover the distance from the source to the receiver inside the material under examination;
- signal propagation velocity, in the sense of the ratio of the distance between source and receiver to transit time;
- signal attenuation characteristics in its passage through the material.

Traditional applications of UT are based on measurements of velocity *V* of acoustic waves propagating through the material. The wave velocity is directly related to a structure's physical parameters, for example, elastic modulus *E*, Poisson's ratio *ν*, and density *d*, so that it is being frequently applied for evaluating structure integrity and effectiveness of restorations. Several efforts have been made to estimate material strength *S* from *V* measurements, starting from the assumption that *V* is directly related to *E*, and *E* is directly related to *S*. However, *S* estimation is acceptable only when a direct correlation between *V* and *S* is available, for example, by measuring *V* in cores extracted from the structure and then tested for *S* determination or by measuring *V* in masonry portions later tested with flat jacks. Nevertheless, it is empirically acknowledged that the higher the strength, the higher the velocity.

Wave velocity measurements are preferentially carried out applying the direct transmission technique (DTT), in which the wave is transmitted by a transducer through the test object and received by a second transducer aligned with the transmitter on the opposite side. This allows measurement of the time *T* that the wave takes to travel through the thickness of the object, from the emitter to the receiver, along a path of length *L*; the average velocity of the wave is simply obtained from the ratio *L*/*T*. The assumption is that the presence of any kind of anomaly (defect, void, and area with different physical-mechanical characteristics) would cause a variation in signal transit time and thus in signal velocity, due to a deviation of the pattern of the wave.

The DTT is very effective, since the broad direction of wave propagation is perpendicular to the source surface and the signal travels through the entire thickness of the item. European standards concerning the determination of wave velocity in structures [19, 20] suggest, therefore, the application of this kind of signal transmission.

The present study aims to attain empirical correlations between *V* and the physical and mechanical properties that describe the behavior of limestone samples. The major significance of the proposed statistical correlations using simple UT consists of the possibility of estimating the mechanical properties of similar limestone litho-types existing in masonry structures of old buildings, especially in the city of Cagliari (Italy). This may be of great value in a preliminary phase of the diagnosis and inspection of the structural and material condition, particularly in cases in which the possibility of sampling material cores is reduced.

## 2. Materials

The buildings of the historical nucleus of the city of Cagliari (Italy) are mainly made of limestone types which are part of the Miocene calcareous lithologic substratum of the city. These materials can be found both as foundation soil and as building material. Their main physical characteristics, drawn from the literature [21], are shown in Table 1.

When the stones have to do with water, problems due to this interaction are increased by litho-type characteristics. Water, in fact, in addition to being an excellent solvent for certain inorganic materials, is able to generate strong mechanical stresses due to volume increases in the transition from liquid to solid. It is also able to carry pollutants, in solution or suspension, which react with the stone, thus encouraging further crumbling. This process in nonporous rocks leads simply to a gradual loss of lithic materials through a thinning of the rock; in porous rocks it generates a dissolution of the grain binder of the stone, which is a process of decohesion. Thus, the mechanical behavior and the level of degradation of the stone masonry are strongly affected by the litho-type properties.

**Figure 1:**
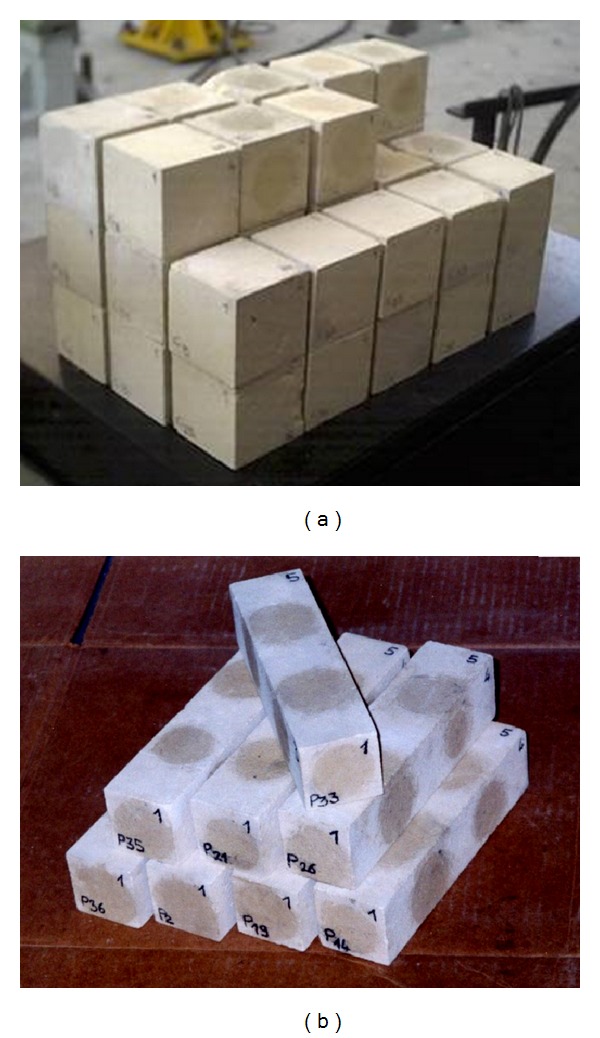
Cubic and prismatic limestone specimens.

The experimental tests were carried out on 38 cubic specimens 0.07 × 0.07 m in size and on 15 prismatic specimens 0.05 × 0.05 × 0.2 m in size (Figure 1), drawn from blocks picked up in one of the quarries active in the past. *V* measurements were carried out and then cubic specimens were tested for compression strength determination, and prismatic ones for elastic modulus determination.

## 3. Experimental Tests

Specimens were weighed and density *d* was determined as *d* = *m*/*v*, where *m* is the weight of the specimen and *v* is its bulk volume [22]. Some rock pieces, debris of block cutting, were weighed before and after a process of (i) heating up to 105°C for 5 days and (ii) cooling down in silica gel. Estimated water content was found to be negligible.

### 3.1. Ultrasonic Testing

UT was carried out on each specimen applying the DTT and *V* measurements were performed.

The testing equipment included

- a Velleman Instruments arbitrary waveform generator for generating signals,
- a pair of piezoelectric transducers (54 kHz resonant frequency) for emitting and receiving signals,
- a Velleman Instruments digital oscilloscope for signals visualization and preliminary analysis,
- a PC for data storage and signal processing.

Transparent vaseline was used to couple the transducers to the sample to reduce signal energy dissipation due to the difference in acoustic impedance between the materials in contact. A support system was arranged specifically for correctly placing the transducers on the specimen.

**Figure 2:**
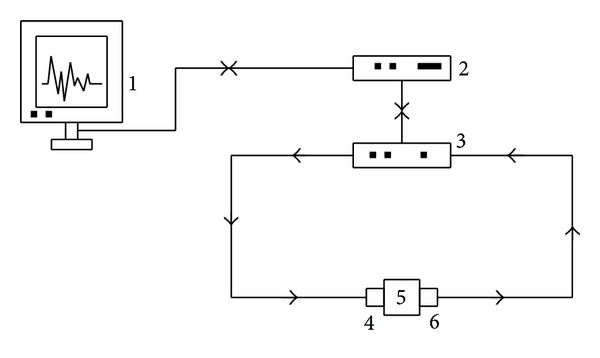
UT setup. (1) PC, (2) oscilloscope, (3) waveform generator, ((4), (6)) transducers, and (5) specimen.

**Figure 3:**
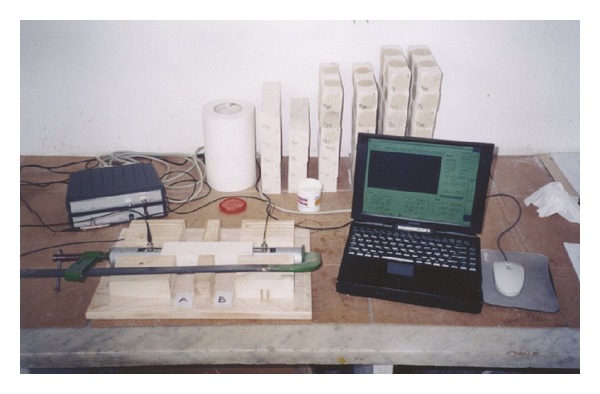
UT operative procedure.

The measurement setup and the operative procedure are shown in Figures 2 and 3, respectively.

### 3.2. Theoretical Background

As efficaciously explained in [23], ultrasonic waves propagate due to the oscillatory motions of particles within a material. An ultrasonic wave may be considered as an infinite number of oscillating particles connected by means of elastic springs. Each individual particle is influenced by the motion of its nearest neighbor and both inertial and elastic restoring forces act upon each particle. A particle on a spring has a single resonant frequency determined by its spring constant *k* and its mass *m*. The spring constant is the restoring force of a spring per unit of length. Within the elastic limit of any material, there is a linear relationship between the displacement of a particle and the force attempting to restore the particle to its equilibrium position. This linear dependency is described by Hooke's Law, which is mathematically written as *F* = −*kx*, where *F* is the force, *k* is the spring constant, and *x* is the amount of particle displacement. The ultrasonic velocity is a function of the properties of the crossed material and it is independent of the amplitude of the wave. As stated by Newton's Second Law, which is mathematically written as *F* = *ma*, the force applied to a particle will be balanced by its mass *m* and acceleration *a*. By considering together Hooke's Law and Newton's Second Law, it can be stated that the applied force and the restoring force are equal, and *ma* = −*kx* can be written. Since mass *m* and spring constant *k* are constants for any given material, it can be inferred that acceleration *a* and displacement *x* are the only variables and that they are directly proportional. It turns out that the time a particle needs to move and return to its equilibrium position is independent of the force applied. So, within a given material, ultrasonic waves always travel at the same speed independently of the applied force when other variables, such as temperature, are held constant.

Ultrasonic waves travel at different speeds in different materials. This is because the mass of the particles and the spring constants are different for different materials. The mass of the particles is related to the density of the material, and the spring constant is related to its elastic constants. The general relationship between the ultrasonic velocity in a solid and its density and elastic constants is given by the following equation:

(1) $$\begin{array}{c} V={\left(\frac{C}{d}\right)}^{1/2}, \end{array}$$

where *V* is the ultrasonic velocity, *C* is the considered elastic constant, and *d* is the material density. This equation may take different forms depending on the type of wave and elastic constants that are used. The typical elastic constants of a material include (i) Young's modulus *E*, (ii) Poisson's ratio *ν*, (iii) Shear modulus *G*, and (iv) Bulk modulus *K*. When the velocity of a longitudinal wave is considered, Young's modulus and Poisson's ratio are commonly used. In that case (1) assumes the form

(2) $$\begin{array}{c} V\;=\;{\left[\frac{\left(E/d\right)\left(1-\nu \right)}{\left(1+\nu \right)\left(1-2\nu \right)}\right]}^{1/2}. \end{array}$$

Thus the relation between *V* and *E*, *ν*, and *d* can be exploited to achieve data regarding the structure's condition in terms of physical and mechanical characteristics.

### 3.3. Mechanical Tests

After UT, cubic specimens were tested to determine their compressive strength (*R*) according to the European standard [24], and prismatic specimens were tested to determine both secant (*E*_*s*_) and tangent (*E*_*t*_) Young's modulus, according to the Italian standard [25].

## 4. Results and Discussion

Experimental results are summarized in Table 2, where subscripts *c* and *p* stand for a parameter measured in cubic or prismatic specimens, respectively.

It can be seen from Table 2 that mechanical properties show a noteworthy high scattering.

### 4.1. Statistical Data Processing

The basis for applying nondestructive measurements is the existence of a relation between the material's characteristics, such as strength, and one or more predictor parameters. The relation must be described mathematically. The deterministic relation cannot be formulated due to its complexity, but it can be established from empirical observations using mathematical statistical methods, usually regression analysis. The regression analysis yields, among other results, the so-called coefficient of correlation *r*, which measures the strength and direction of a linear relationship between two variables. The value of *r* is such that −1 < *r* < +1. A correlation greater than 0.8 is generally described as strong, whereas a correlation of less than 0.5 is generally described as weak, but these values can vary based upon the type of data being examined.

The correlation between *V* and *d*, *R*_*c*_, *E*_*s*_, and *E*_*t*_, respectively was evaluated, and results are reported in Table 3 in terms of coefficient of correlation *r*.

As can be inferred from Table 3, the correlation coefficients are remarkably high, thus suggesting a strong correlation between ultrasonic velocity and the other parameters.

The significance of the *r*-values can be determined with the *t*-test, assuming that all the variables are normally distributed and the observations are chosen randomly. The test compares the computed *t*-value to a tabulated *t*-value for the specific level of confidence using the null hypothesis. In this test, a 95% level of confidence was chosen. If the computed *t*-value is greater than the tabulated *t*-value, the null hypothesis is rejected, meaning that *r* is significant. Otherwise, if the computed *t*-value is less than the tabulated *t*-value, the null hypothesis is not rejected.

Table 4 shows the results of the *t*-test for the evaluated correlations.

As can be seen in Table 4, all the computed *t*-values remain in the critical upper region, thus leading to the conclusion that there is a real correlation between ultrasonic velocity and the other physical and mechanical parameters, thus supporting the engineering use of the correlations.

When performing regression analysis, the coefficient of determination *r* ^2^ is also useful because it gives the proportion of the variance of one variable that is predictable from the other variables. The coefficient of determination is the ratio of the explained variation to the total variation. It is such that 0 < *r* ^2^ < 1 and denotes the strength of the linear association between the two variables involved. It represents the percent of the data that is the closest to the line of best fit, thus being a measure of how well the regression line represents the data. If the regression line passes exactly through every point on the scatter diagram, it would be able to explain all of the variation. The further the line is away from the points, the less it is able to explain.

**Figure 4:**
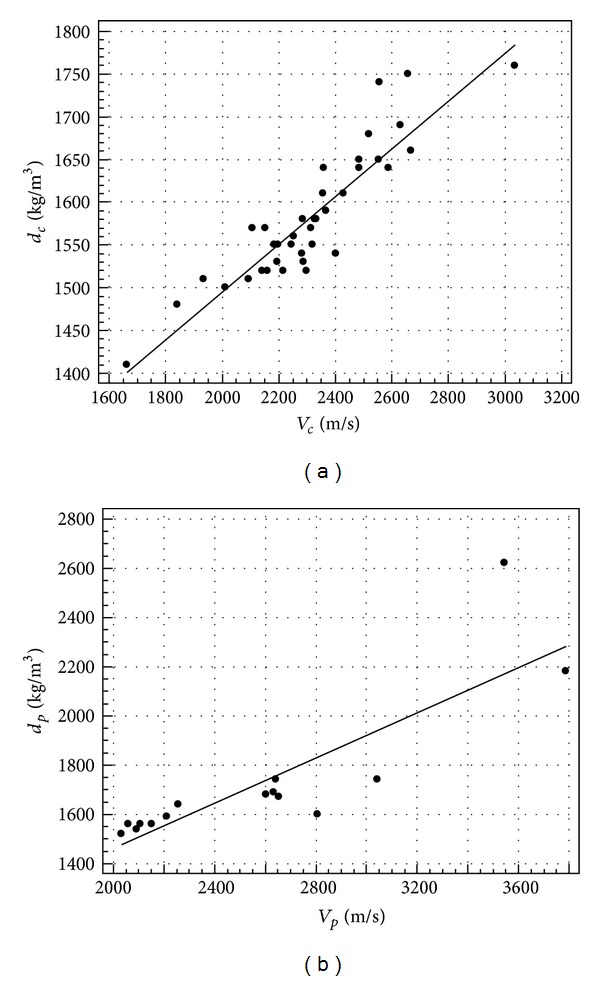
Regression of ultrasonic velocity and density in cubes (a) and prisms (b).

**Figure 5:**
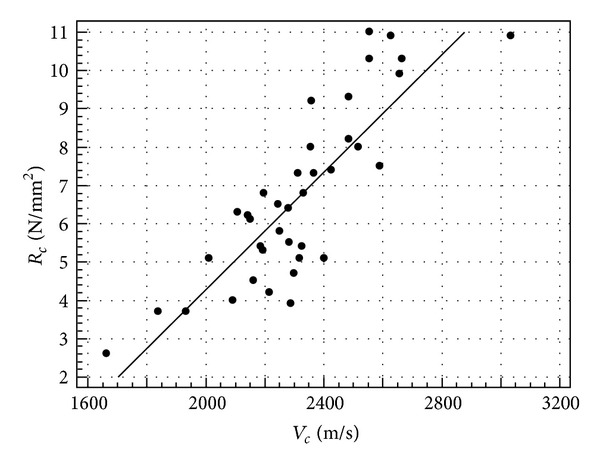
Regression between ultrasonic velocity and compressive strength in cubes.

**Figure 6:**
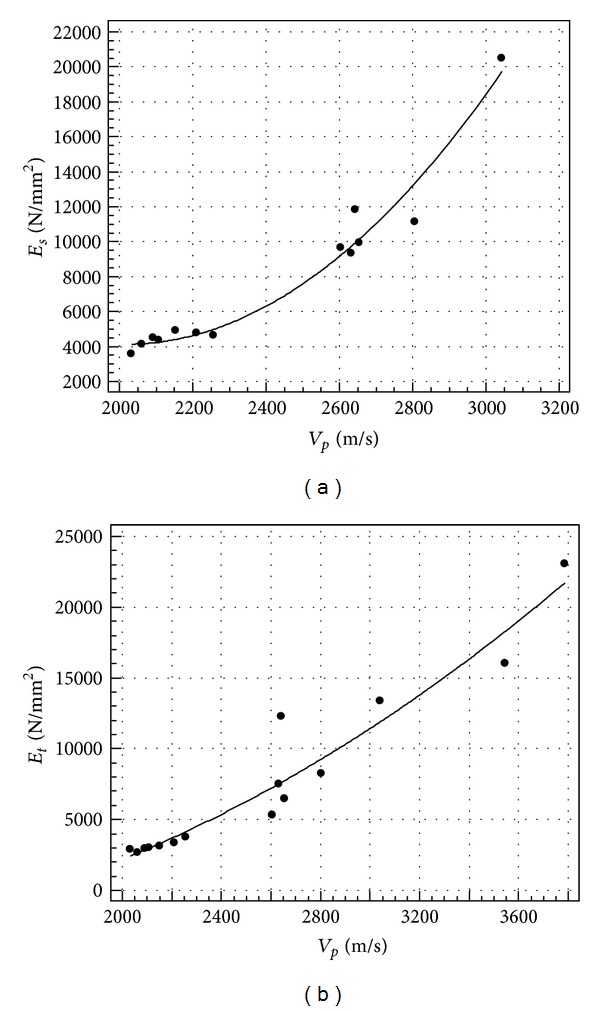
Regression between ultrasonic velocity and secant (a) and tangent (b) elastic modulus.

The results of the regression analysis of *V* to *d*, *R*_*c*_, *E*_*s*_, and *E*_*t*_, respectively, are shown in Figures 4, 5, and 6 in terms of scatter diagram and regression line, while Table 5 reports the regression equations and the related coefficient of determination *r* ^2^.

The analysis of variance for the regression was employed to test the significance of the linear regressions. In this test, which follows a *F*-distribution, a 95% level of confidence was chosen. If the computed *F*-value is greater than the tabulated *F*-value for the specific degree of freedom, then the null hypothesis is rejected, meaning that there is a real relation between the variables.

Analyses of variance for the test regressions are given in Table 6.

From Table 5, it can be seen that, for all the predicted parameters, an *r* ^2^ value higher than 0.7 is reached, which means that more than 70% of parameter variability can be explained by ultrasonic velocity. The modulus of elasticity is clearly the better predicted parameter. For this reason, it is worth noting that theoretically the propagation velocity depends on the dynamic modulus of elasticity of the continuum medium (see (1)).

From Table 6, it can be inferred that all the computed *F*-values for the test procedures are greater than the tabulated ones, meaning that in all cases the null hypothesis is rejected and a clear relation between *V* and the other variables was found.

In order to evaluate further the goodness of fit of the selected regression models, the analysis of residuals was performed. Residuals are the differences between the predicted values and the observed values for the dependent variable. Residuals are commonly represented as a scatter diagram, the so-called residuals plot. A residuals plot is a graph that shows the residuals on the vertical axis and the independent variable (predictor) on the horizontal axis. If the points in a residuals plot are randomly dispersed around the horizontal axis, the regression model is appropriate for the data. If the residuals display a certain pattern, a different regression model should be selected, because a trend would indicate that the residuals were not independent.

**Figure 7:**
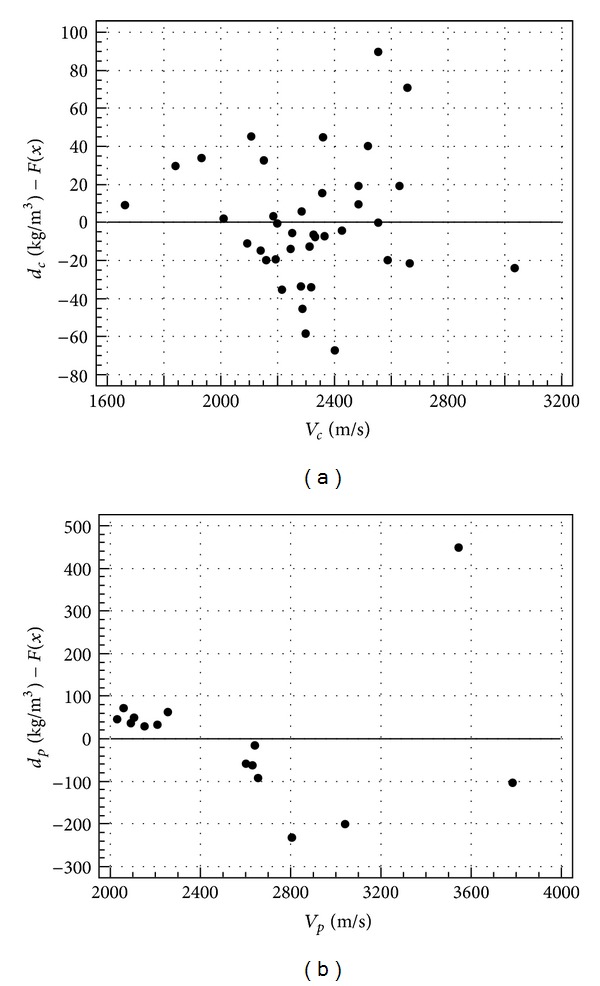
Residuals plot of density in cubes (a) and prisms (b).

**Figure 8:**
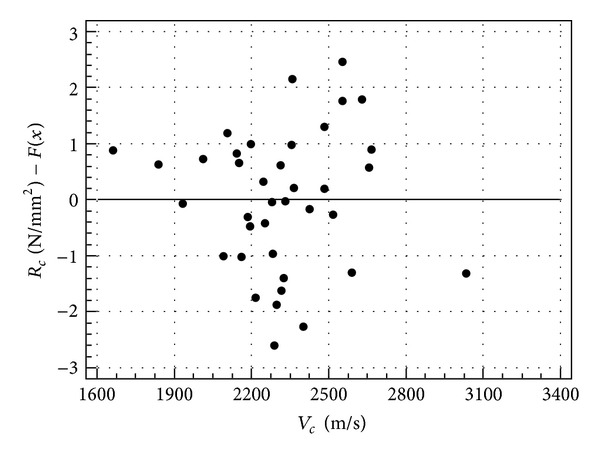
Residuals plot of compressive strength in cubes.

**Figure 9:**
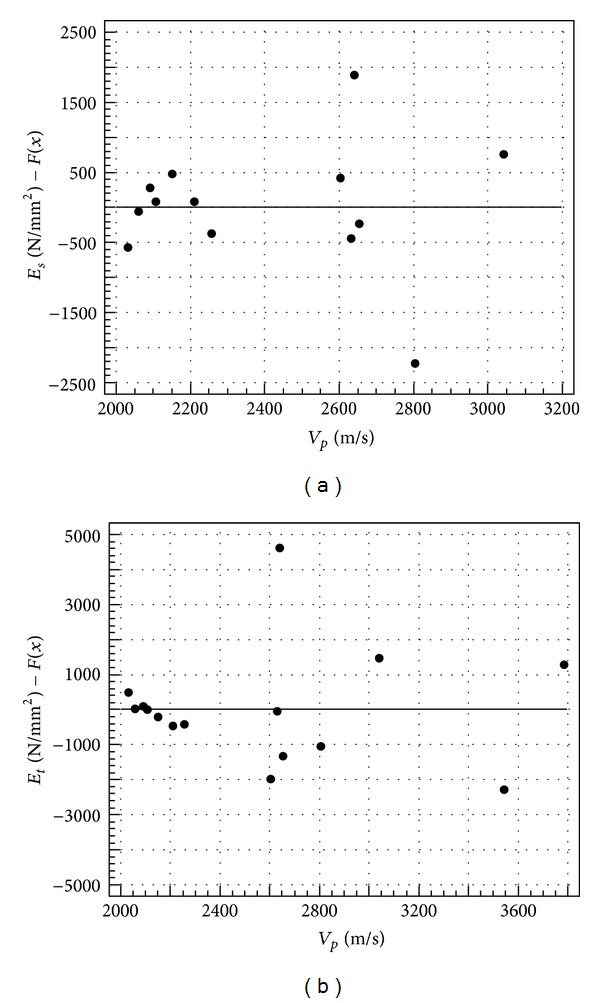
Residuals plot of secant (a) and tangent (b) elastic modulus.

Residuals plots related to the regression models reported in Table 5 are shown in Figures 7, 8, and 9.

It can be noted that the scatter diagrams are disorderly and the residuals do not show any trend, meaning that the regression models are appropriate and *V* may be considered as a good predictor of the analyzed physical and mechanical properties.

## 5. Conclusions

In this paper, the feasibility of ultrasonic velocity in predicting some physical and mechanical features of building limestone is analyzed. Several limestone specimens were tested and correlation between ultrasonic velocity *V* and density, compressive strength, and modulus of elasticity is presented and discussed.

The effectiveness of ultrasonic velocity was evaluated by regression, with the aim of observing the coefficient of determination *r* ^2^ between *V* and the aforementioned parameters. The coefficient of determination was used as a measure of the ability of the parameter *V* to predict the other properties of the materials. The coefficients of determination for the regressions performed were remarkably high, thus suggesting a significant correlation between the variables involved in engineering use. This correlation, as well as the predictive capacity of ultrasonic velocity, was strengthened by the results of residuals analysis. The strong relations that were established between ultrasonic velocity and the limestone properties, such as density, compressive strength, and modulus of elasticity, indicate that these parameters can be reasonably estimated by means of this nondestructive parameter.

Results achieved may be of practical significance for the conservation, rehabilitation, and strengthening of stone masonry buildings. These structures form an integral part of the historical building heritage in Europe and throughout the world. When dealing with preservation and restoration of this kind of buildings, it is necessary to assess their serviceability and, if possible, their load-carrying capacity. Assessment of such buildings can however be difficult as there is little knowledge or experience available on their design, and the evaluation of their real state should not interfere with the condition and functionality of the building and should possibly involve limited costs. To provide confidence for the result of the assessment, reliable input parameters are required and effective inspection and measurement methods are necessary to establish or verify the input parameters. Destructive testing of blocks and walls would be the most reliable mechanism for evaluation of mechanical parameters, such as compressive strength, and quality of masonry structures. However, this approach calls for a large number of specimens to obtain statistically valid results, and it is a very laborious, time- and money-consuming, and invasive task. In this frame, an alternative solution for estimating masonry properties by nondestructive testing should be searched for.

## Figures and Tables

**Table 1:** Main physical characteristics of local limestone.

	Apparent specific gravity (kg/m^3^)	Real specific gravity (kg/m^3^)	Dry density (kg/m^3^)	Porosity (%)	CaCO_3_ content (%)
Mean	1982	2485	1985	7.15	81.5
St. dev.	0.09	0.05	0.15	0.9	12.3

**Table 2:** Statistical summary of the experimental results.

	Average	Standard deviation (%)
*d*_*c*_ (kg/m^3^)	1580	4.87
*d*_*p*_ (kg/m^3^)	1680	9.64
*R*_*c*_ (N/mm^2^)	6.7	33.97
*E*_*s*_ (N/mm^2^)	7930	40.98
*E*_*t*_ (N/mm^2^)	7548	46.89
*V*_*c*_ (m/s)	2315	10.77
*V*_*p*_ (m/s)	2517	18.50

**Table 3:** Coefficient of correlation *r* between *V* and physical-mechanical parameters.

	*V*_*c*_	*V*_*p*_
*d*_*c*_	0.9052	
*d*_*p*_		0. 8494
*R*_*c*_	0.8394	
*E*_*s*_		0. 9440
*E*_*t*_		0. 9572

**Table 4:** Results of the *t*-test for the evaluated correlations.

Variable	*t*-test	*t*-table
*d*_*c*_	17.28	2.00
*d*_*p*_	5.33	2.05
*R*_*c*_	57.05	2.00
*E*_*s*_	4.27	2.05
*E*_*t*_	3.10	2.05

**Table 5:** Regression equations and coefficient of determination *r* ^2^ of *V* to physical and mechanical parameters.

Regression equations	Coeff. of determination *r* ^2^
*d*_*c*_ = 935.56 + 0.28*V*_*c*_	0.8193
*d*_*p*_ = 538.49 + 0.46*V*_*p*_	0.7214
*R*_*c*_ = −11.0333 + 0.01*V*_*c*_	0.7045
*E*_*s*_ = 64265.6687 − 59.6509*V*_*p*_ + 0.0148*V*_*p*_^2^	0.9632
*E*_*t*_ = −3419.1747 − 1.4909*V*_*p*_ + 0.002*V*_*p*_^2^	0.9267

**Table 6:** Results of the *F*-test for the analysis of variance.

Variable	*F*-test	*F*-table
*d*_*c*_	163.25	4.17
*d*_*p*_	33.66	4.67
*R*_*c*_	85.83	4.17
*E*_*s*_	288.03	4.67
*E*_*t*_	164.31	4.67

## References

[1] McCann DM, Forde MC (2001). Review of NDT methods in the assessment of concrete and masonry structures. *NDT and E International*.

[2] Hoła J, Schabowicz K (2010). State-of-the-art non-destructive methods for diagnostic testing of building structures—anticipated development trends. *Archives of Civil and Mechanical Engineering*.

[3] Bungey JH, Millard SG, Grantham MG (2006). *Testing of Concrete in Structures*.

[4] Krautkramer J, Krautkramer H (1990). *Ultrasonic Testing of Materials*.

[5] Berke M Nondestructive material testing with ultrasonics—introduction to the basic principles. http://www.ndt.net.

[6] Berra M, Binda L, Baronio G, Faticcioni A Ultrasonic pulse transmission: a proposal to evaluate the efficiency of masonry strengthening by grouting.

[7] Cannas B, Carcangiu S, Fanni A (2013). Frequency analysis of ultrasonic signals for non-destructive diagnosis of masonry structures. *Advances in Civil Engineering and Building Materials*.

[8] Cannas B, Carcangiu S, Fanni A (2013). Ultrasonic testing of masonry structures by features extraction and self organising maps. *Advances in Civil Engineering and Building Materials*.

[9] Camplani M, Cannas B, Carcangiu S (2008). Acoustic tomography for non destructive testing of stone masonry. *Computational Science and Its Applications*.

[10] Camplani M, Cannas B, Cau F, Concu G, Usai M (2008). Acoustic NDT on building materials using features extraction techniques. *Computational Science and Its Applications*.

[11] Agarwal R, Singh VR (2000). Ultrasonic parameters and relationship between compressive strength, microstructure of gall bladder stones. *European Journal of Ultrasound*.

[12] Yasar E, Erdogan Y (2004). Correlating sound velocity with the density, compressive strength and Young’s modulus of carbonate rocks. *International Journal of Rock Mechanics and Mining Sciences*.

[13] Gaviglio P (1989). Longitudinal waves propagation in a limestone: the relationship between velocity and density. *Rock Mechanics and Rock Engineering*.

[14] Khandelwal M, Singh TN (2009). Correlating static properties of coal measures rocks with P-wave velocity. *International Journal of Coal Geology*.

[15] Mahure NV, Vijh GK, Sharma P, Sivakumar N, Ratnam M (2011). Correlation between pulse velocity and compressive strength of concrete. *International Journal of Earth Sciences and Engineering*.

[16] Voigt T, Akkaya Y, Shah SP (2003). Determination of early age mortar and concrete strength by ultrasonic wave reflections. *Journal of Materials in Civil Engineering*.

[17] Kewalramani MA, Gupta R (2006). Concrete compressive strength prediction using ultrasonic pulse velocity through artificial neural networks. *Automation in Construction*.

[18] Bogas JA, Gomes MG, Gomes A (2013). Compressive strength evaluation of structural lightweight concrete by non-destructive ultrasonic pulse velocity method. *Ultrasonics*.

[19] EN 12504-4 (2004). *Testing Concrete- Part 4. Determination of Ultrasonic Pulse Velocity*.

[20] EN 14579 (2004). *Natural Stone Test Methods. Determination of Sound Speed Propagation*.

[21] Barrocu G, Crespellani T, Loi A (1981). Caratteristiche geologico-tecniche del sottosuolo dell’area urbana di Cagliari. *Rivista Italiana di Geotecnica*.

[22] EN 1936 (2007). *Natural Stone Test Methods—Determination of Real Density and Apparent Density, and of Total and Open Porosity*.

[23] NDT Resource Center Basic Principles of Ultrasonic Testing. http://www.ndt-ed.org/EducationResources/CommunityCollege/Ultrasonics/cc_ut_index.htm.

[24] EN 1926 (2006). *Natural Stone Test Methods—Determination of Uniaxial Compressive Strength*.

[25] UNI 9724/8 (1992). *Materiali Lapidei—Determinazione del Modulo Elastico in Compressione*.

